# The presence of cortical neurons in striatal-cortical co-cultures alters the effects of dopamine and BDNF on medium spiny neuron dendritic development

**DOI:** 10.3389/fncel.2015.00269

**Published:** 2015-07-20

**Authors:** Rachel D. Penrod, Justin Campagna, Travis Panneck, Laura Preese, Lorene M. Lanier

**Affiliations:** ^1^Department of Neuroscience, University of MinnesotaMinneapolis, MN, USA; ^2^Graduate Program in Neuroscience, University of MinnesotaMinneapolis, MN, USA

**Keywords:** medium spiny neurons (MSN), striatum, *in vitro*, development, dendritic spines, dendritic branching, dopamine, BDNF

## Abstract

Medium spiny neurons (MSNs) are the major striatal neuron and receive synaptic input from both glutamatergic and dopaminergic afferents. These synapses are made on MSN dendritic spines, which undergo density and morphology changes in association with numerous disease and experience-dependent states. Despite wide interest in the structure and function of mature MSNs, relatively little is known about MSN development. Furthermore, most *in vitro* studies of MSN development have been done in simple striatal cultures that lack any type of non-autologous synaptic input, leaving open the question of how MSN development is affected by a complex environment that includes other types of neurons, glia, and accompanying secreted and cell-associated cues. Here we characterize the development of MSNs in striatal-cortical co-culture, including quantitative morphological analysis of dendritic arborization and spine development, describing progressive changes in density and morphology of developing spines. Overall, MSN growth is much more robust in the striatal-cortical co-culture compared to striatal mono-culture. Inclusion of dopamine (DA) in the co-culture further enhances MSN dendritic arborization and spine density, but the effects of DA on dendritic branching are only significant at later times in development. In contrast, exogenous Brain Derived Neurotrophic Factor (BDNF) has only a minimal effect on MSN development in the co-culture, but significantly enhances MSN dendritic arborization in striatal mono-culture. Importantly, inhibition of NMDA receptors in the co-culture significantly enhances the effect of exogenous BDNF, suggesting that the efficacy of BDNF depends on the cellular environment. Combined, these studies identify specific periods of MSN development that may be particularly sensitive to perturbation by external factors and demonstrate the importance of studying MSN development in a complex signaling environment.

## Introduction

Medium spiny neurons (MSNs) are the primary neuron type of the striatum and are morphologically identified by their cell body size, dendritic arborization pattern and high density of dendritic spines (Kemp, 1968; Graveland and DiFiglia, 1985; Rafols et al., 1989; Matamales et al., 2009). In addition to its involvement in neurodegenerative disorders such as Parkinson’s and Huntington’s diseases, anomalies in the striatum and its basal ganglia connections have been implicated in neurodevelopmental disorders including autism, Tourette’s, schizophrenia, obsessive-compulsive disorders (OCD) and attention-deficit/hyperactivity disorder (ADHD; Pappas et al., 2014). Strikingly, alterations in MSN dendritic arborization are the primary morphological abnormality observed with deletions of genes associated with Tourette’s, OCD and autism (Shmelkov et al., 2010; Bacon and Rappold, 2012). Because the organization of dendrites affects the formation and efficacy of synapses and impacts the transmission of signals back to the soma, developmental events that regulate dendritic arborization and spine formation could have a large impact on neural signaling (Kulkarni and Firestein, 2012).

Dendritic arborization is shaped by a combination of cell-intrinsic and extrinsic factors (Jan and Jan, 2003). In the developing striatum, signals from nascent dopaminergic and glutamatergic afferents play important roles in MSN development. Dopaminergic afferents arise from the substantia nigra pars compacta (SNc) and are believed to reach the striatum around embryonic day 14 (E14) in the rat (roughly equivalent to E12 in mouse; Voorn et al., 1986, 1988). Additionally, striatal expression of dopamine (DA) receptors is detected as early as E14 in rat, with expression levels reaching approximately 75% that of an adult by birth (Schambra et al., 1994). Glutamatergic corticostriatal fibers are believed to be collaterals of corticofugal axons that branch into the striatum as early as E18 in the rat (Nisenbaum et al., 1998; Sheth et al., 1998). In addition to releasing neurotransmitters, both the nigrostriatal and corticostrioatal projections are thought to release brain-derived neurotrophic factor (BDNF; Baydyuk and Xu, 2014). Developmental alterations in signaling by DA, glutamate, or BDNF have been shown to affect MSN dendritic arborization *in vivo* (Baydyuk et al., 2011; Beutler et al., 2011; Cazorla et al., 2014); however, elucidation of the mechanisms involved and the interactions among these signaling pathways has been difficult *in vivo*.

A great deal of previous *in vitro* research into mammalian dendrite development has focused on pyramidal neurons, a common subtype of excitatory neuron in the hippocampus and neocortex. Although MSNs are very different from pyramidal neurons in terms of morphology (symmetric vs. asymmetric dendrites; Horton et al., 2005), embryological origin (ganglionic eminence vs. cortical sub-ventricular zone (SVZ); Angevine and Sidman, 1961; Wichterle et al., 1997), transmitter release (GABA vs. glutamate), excitability (long vs. short latency firing; Murer et al., 2002), and projection patterns, the assumption has been that MSNs have similar dendritic growth patterns. If, in contrast, MSNs differ in terms of the molecular determinants of their morphological development, these cell-type specific mechanisms may be at least a partial explanation for why these neurons are differentially affected in multiple neurological disorders. Clearly, understanding the developmental profiles of MSNs, instead of generalizing from distantly related pyramidal neurons, will be a key step in understanding the striatum and its distinctive vulnerability to neurological disease.

Commonly used protocols for culturing MSNs have historically relied on striatal mono-culture (e.g., Ventimiglia and Kindsay, 1998), a system that yields high numbers of MSNs that retain a relatively simple morphology with low densities of dendritic spines, unlike those observed *in vivo*. In striatal mono-culture, the lack of dendritic structures similar to those found *in vivo* complicates efforts to study dendritic arbors, spines, and the molecules that regulate them. Compared to striatal mono-culture, MSNs co-cultured with glutamatergic neurons develop more complex dendritic arbors and higher densities of dendritic spines, similar to those *in vivo* (Segal et al., 2003; Tian et al., 2010; Penrod et al., 2011). Here, we have examined the development of MSN dendrite arbor, spine density, and spine morphology using a striatal-cortical culture system and demonstrate that the timing and extent of MSN response to DA and BDNF is dependent on the complexity of the environment.

## Materials and Methods

### Animals

Animal procedures were performed at the University of Minnesota in facilities accredited by the Association for Assessment and Accreditation of Laboratory Animal Care (AAALAC) and in accordance with protocols approved by the University of Minnesota IACUC, as well as the principles outlined in the National Institute of Health *Guide for the Care and Use of Laboratory animals*.

### Cell Culture

**Figure 1:**
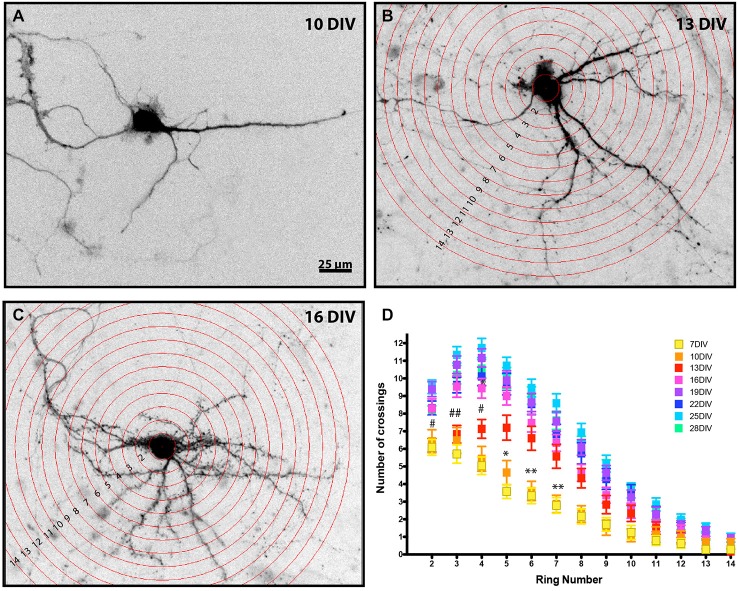
**Developmental analysis of MSN dendritic complexity.** Representative images of enhanced green fluorescent protein (EGFP) filled MSNs at **(A)** 10 DIV, **(B)** 13 DIV, and **(C)** 16 DIV (Sholl rings are overlaid in **B**, **C**). Images have been inverted and background modified for presentation. **(D)** Sholl analysis of DARPP32+ MSNs from 7–28 DIV. MSNs undergo a time-dependent increase in dendrite branching. (*) and (#) represent significant difference from the preceding time point: (*) is 10–13 DIV and (#) is 13–16 DIV. *or ^#^*p* < 0.05, **or ^##^*p* < 0.01. Data displayed as Mean ± SEM. *N* = 23–48 neurons, depending on time point. Scale bar in **(A)** = 25 μm.

**Figure 2:**
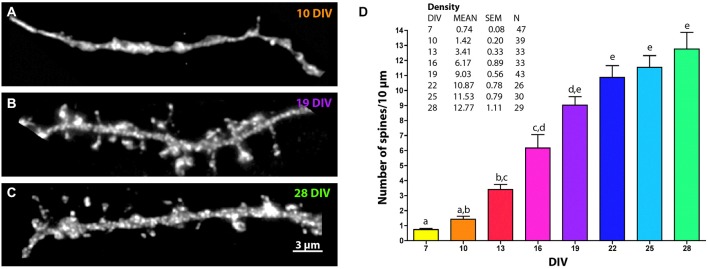
**Changes in MSN spine density across development.** Representative images of EGFP-filled MSN terminal tip dendrites at **(A)** 10 DIV, **(B)** 19 DIV and **(C)** 28 DIV. **(D)** Quantification of spine density over time. Letters represent time points that are not significantly different from one another. Spine density increased from 7 to 13 DIV (*p* < 0.01), from 10 to 16 DIV (*p* < 0.01), from 13 to 19 DIV (*p* < 0.001), from 16 to 22 DIV (*p* < 0.05), and was statistically stable from 19 DIV onward. Data displayed as Mean ± SEM. *N* = 26–47 neurons, depending on time point. Scale bar in **(C)** = 3 μm.

**Figure 3:**
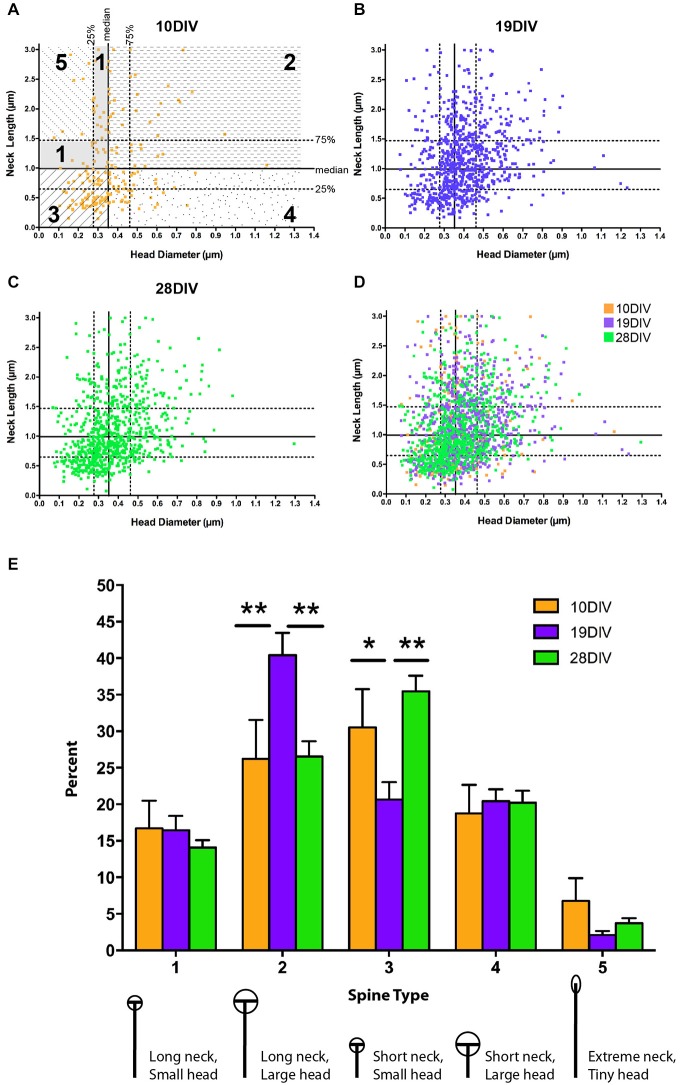
**The relationship between MSN spine head diameter (HD) and neck length (NL) is highly variable across development. (A–D)** Scatter plots of paired HD (x-axis) and NL (y-axis) measurements for spines at **(A)** 10 DIV, **(B)** 19 DIV, **(C)** 28 DIV, and **(D)** all three time-points simultaneously. Medians are determined from the entire population (7–28 DIV) and displayed as solid lines with 75^th^ and 25^th^ percentiles as dotted lines in **(A)**. Portions of the graph that reflect different spine types are marked and labeled as 1–5. **(E)** Using a median split spine classification scheme based on observed MSN spine morphologies across development, a transient increase in type 2 spines partnered with a transient decrease in type 3 spines is detected at 19 DIV. *p < 0.05, **p < 0.01. Morphologies are diagrammed and described below each type. Values are reported as percent of type per segment. Data displayed as Mean ± SEM. *N* = 31–45 segments, depending on DIV.

**Figure 4:**
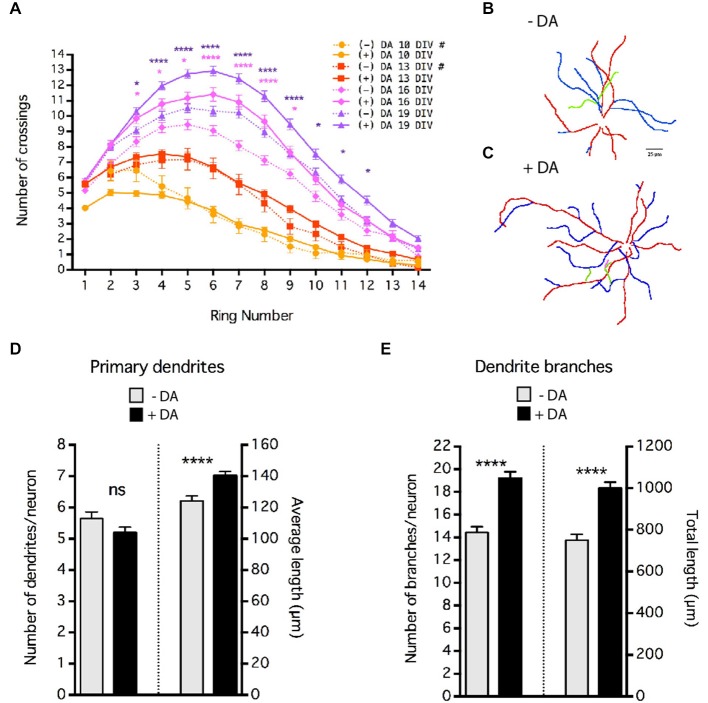
**Dopamine (DA) regulation of MSN dendrite arborization.** Striatal-cortical co-cultures were grown in the absence (−DA) or presence (+DA) of 1 μM DA. **(A)** Sholl analysis of dendritic complexity of MSNs grown in the presence (+DA) or absence (−DA) of DA. # indicates that data for 10 and 13 DIV (–)DA time course are taken from Figure 1D. Significance was determined only for 16 and 19 DIV (−) vs. (+) DA. **(B,C)** Examples of neurite tracing of 19 DIV MSNs grown in the absence **(B)** or presence **(C)** of 1 μM DA. Dendrites and branches are color-coded (primary dendrite (red), secondary branch (blue), tertiary branch (green), quaternary branch (pink)). **(D)** Quantification of the number and length of primary dendrites. **(E)** Quantification of the number of braches and total branch length per neuron. For **(A)**, **(D)** and **(E)** *p < 0.05, **p < 0.01, ***p < 0.001, ****p < 0.0001. Data displayed as Mean ± SEM. *N* = 70–138 neurons for each treatment. Scale bar in **(B)** = 25 μm.

**Figure 5:**
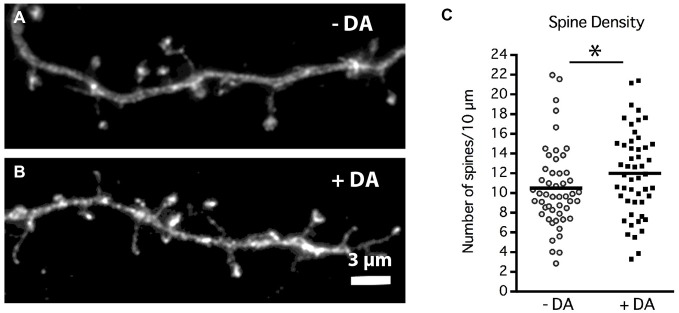
**DA regulation of MSN dendritic spine density.** Striatal-cortical co-cultures were grown in the absence (−DA) or presence (+DA) of 1 μM DA from 4–19 DIV. **(A,B)** Representative examples of dendrite segments from neurons grown in the **(A)** absence or **(B)** presence of DA. **(C)** Scatter plot of the mean number of spines per 10 μM of dendrite shaft for each neuron. Population mean is represented by the solid horizontal bar (10 ± 0.58 control vs. 12 ± 0.61 spines/10 μm with DA). *p < 0.05. *N* = 50 neurons for each treatment. Scale bar in **(B)** = 3 μm.

**Figure 6:**
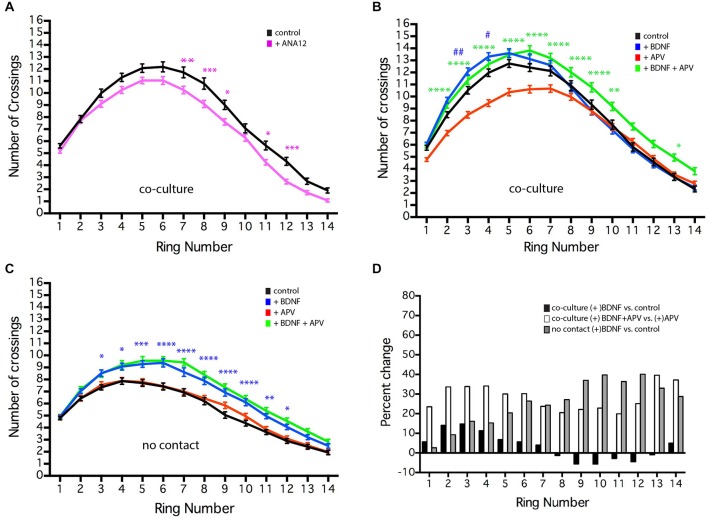
**NMDAR-dependent effects of BDNF of MSN dendritic arborization.** MSNs were grown in the presence or absence of BDNF (10 ng/ml), APV (50 μm), or ANA12 (10 μM) from 4–19 DIV. **(A)** Sholl analysis of control (black line) cultures or cultures treated with ANA12 (pink line). **(B)** Sholl analysis of co-cultures under various conditions: untreated control (black line), treated with BDNF (blue line), APV (red line) or BDNF + APV (green line). Symbols indicate significant differences: (#) is +BDNF vs. control, (*) = is +BDNF+APV vs. +APV. **(C)** Sholl analysis of no-contact control (black line) or no-contact cultures treated with BDNF (blue line), APV (red line) or BDNF + APV (green line). Symbols represent significant difference from the control: (*) = + BDNF vs. control. *or ^#^*p* < 0.05, **or ^##^*p* < 0.01, ***p < 0.001, ****p < 0.0001. Data displayed as Mean ± SEM. *N* = 85–119 neurons for each treatment. **(D)** To facilitate comparisons, values for the contact + BDNF ± APV and the no-contact + BDNF conditions are plotted as a percentage of their respective controls. This illustrates that in the absence of NMDAR activity (gray and white bars), BDNF significantly increases branching, while in the presence of NMDAR activity, the effects of BDNF are limited.

Primary striatal-cortical cultures were prepared as previously described (Penrod et al., 2011). Briefly, the ganglionic eminence (presumptive striatum) and prefrontal cortex were removed from day 16 mouse embryos. Tissues were separately digested at 37°C for 15–30 min. in a final concentration of 0.25% trypsin-EDTA (Sigma-Aldrich, T4174), rinsed briefly in calcium magnesium free Hanks balanced salts (CMF-HBSS), and resuspended in neuronal plating media (10 mM HEPES, 10 mM sodium pyruvate, 0.5 mM glutamine, 12.5 μM glutamate, 10% Newborn Calf Serum, 0.6% glucose in minimal essential media plus Earl’s salt (EMEM)). Tissues were separately dissociated by trituration with a fire-polished pipette. Following dissociation, cells were counted using trypan blue and a hemocytometer. Cells were plated in 35 mm dishes containing five 12 mm acid-washed glass coverslips coated with 100 μg/ml poly-d-lysine/4 μg/ml laminin, and filled with neuronal plating media. Cells were plated in a 3:2 cortex to striatum ratio at a final cell density of 200 cells/mm^2^. Dishes were maintained in a 37°C, five percent CO_2_ incubator. After the cells adhered (1–3 h after plating), plating media was replaced with growth media (Neurobasal, 1 × B27 (Invitrogen), 0.5 mM glutamine) pre-conditioned on glia (see below). For data in Figures 1–3, 50% of the media was removed and replaced with fresh glia-conditioned media on days 7 and 14. However, by chance we discovered that although glia conditioned media is important for the initial stages of neurite initiation and outgrowth, prolonged use of glia conditioned media after 4 days *in vitro* (DIV) reduces overall health of the cultures after 14 DIV (about the time that dendritic spines and synapses begin to form). Therefore, for subsequent experiments (Figures 4–6), cultures were kept in glia-conditioned media until 4 DIV, when the glia-conditioned media was replaced with fresh, unconditioned growth media. On 7 DIV and every week thereafter, half of the media was replaced with fresh, unconditioned growth media. These changes likely account for the fact that in Figure 4 growth of the controls at 16 and 19 DIV is more robust than seen at the same time points in Figure 1.

For no-contact experiments in which striatal and cortical cells are grown in the same dish but physically separated, small wax drops were attached to 25 mm acid-washed glass coverslips prior to poly-d-lysine and laminin coating. Cortical cells were plated alone onto these coverslips at the same density as in co-culture. Following the adhering period, wax-drop coverslips containing cortical cells were inverted into 35 mm dishes containing five 12 mm acid-washed coverslips with only striatal cells plated at the same density as in co-culture.

For experiments with DA, BDNF, 2-amino-5-phosphonovaleric acid (APV), or ANA12, 1 μm DA, 10 ng/ml BDNF, 50 μm APV or 10 μm ANA12 was added during the media change at 4 DIV. All drugs were administered chronically, meaning after initial addition, there is continual exposure without removal of pre-existing drug within the dish. To compensate for drug depletion by breakdown and/or cell uptake, media was supplemented with fresh drug (final concentration 1 μM DA, 10 ng/ml BDNF, 50 μm APV or 10 μm ANA12, assuming total breakdown) every 3–4 days. However, variation of actual concentration within the dish is likely. Experiments were conducted on cultures ranging in age from 7 to 28 DIV.

Glia cultures were prepared as previously described (Penrod et al., 2011). Briefly, the cortices of postnatal (P1–2) mice were removed, chopped into small pieces and incubated with 0.25% trypsin-EDTA and 1 μl/ml Benzonase (Novagen) or 3 mg/ml DNAse1 (Sigma) for 30 min at 37°C. After incubation, an equal volume of glia plating media (10 mM HEPES, 1 mM sodium pyruvate, 2 mM glutamine, 10% Newborn Calf Serum, 0.6% Glucose, 1 × Penicillin-Streptomycin) was added and cells were collected by centrifugation (1000 g for 2 min). Tissue was resuspended in glia plating media, triturated using a flame-polished glass pipette, and filtered through a 0.7 μm cell strainer. Cells were plated on uncoated 10 cm tissue culture dishes. Glia media was replaced 1 day after plating and once per week each subsequent week. Glia-conditioned media was prepared by incubating 10 ml of neuron growth media on confluent glial plates for 48 h. Once the conditioning period was complete, conditioned media was removed and replaced with fresh glia plating media.

### Neuronal Transfection

For enhanced green fluorescent protein (EGFP) expression, the striatal cell population was electroporated prior to plating using the Lonza Nucleofector system and the mouse neuron transfection reagent. For transfection, 1 × 10^6^ dissociated striatal cells were centrifuged at 1000 g for 5 min, plating media was removed, and cells were resuspended in 100 μl of complete transfection reagent containing 10 μg of pCAG-EGFP plasmid (EGFP under the chicken beta-actin promoter with a CMV enhancer). Immediately following electroporation, cells were moved into 2 ml of pre-warmed plating media. After a short (less than 2 min) equilibration period, cells were counted using a hemocytometer and trypan blue to determine viability.

### Immunofluorescence

At designated time points, coverslips were fixed at 4°C with four percent paraformaldehyde/PHEM (60 mM PIPES pH 7.0, 25 mM K-HEPES pH 7.0, 10 mM EGTA, 2 mM MgCl_2_)/0.12 M sucrose-buffered fixative for 15–20 min. Following fixation, cells were rinsed in 1 × PBS and blocked in three percent bovine serum albumin (BSA)/phosphate-buffered saline (PBS) for 30 min at room temperature or 4°C overnight prior to permeabilization. Cells were permeabilized using 0.2% Triton X-100 in PBS for 10 min at room temperature, rinsed in 1 × PBS, and blocked for at least 15 min in three percent BSA/PBS prior to staining. All antibody mixtures were prepared in one percent BSA/PBS and coverslips were incubated overnight at 4°C with primary antibody mixture. All antibody mixtures contained polyclonal rabbit anti Dopamine and cAMP regulated phospho-protein of 32 KDa (DARPP-32; Cell signaling, cat. #2302, 1:250) in order to identify MSNs. To examine dendritic spine morphology, coverslips were stained with DARPP-32 and mouse monoclonal EGFP (Invitrogen, cat. #A11120, 1:1000). Following overnight primary incubation, coverslips were rinsed in 1 × PBS and incubated in secondary antibody mixture for 1 h at room temperature. The following secondary antibodies were used: donkey anti-rabbit conjugated to Texas Red or TRITC, donkey anti-mouse conjugated to FITC (Jackson Immunoresearch). All secondaries were used at 1:100. Following secondary incubation, coverslips were rinsed in 1 × PBS and mounted on glass slides with 2.5% 1,4-Diazabicyclo-[2.2.2]Octane, 150 mM Tris pH 8.0, and 80% glycerol mountant to reduce photobleaching.

### Imaging

For neurite tracing and Sholl analysis (Sholl, 1953), images were collected using Openlab software (Improvision/Perkin Elmer) and a 20X objective on a Zeiss Axiovert 200M microscope. Image analysis was conducted using ImageJ (NIH). Isolated MSNs were identified using DARPP-32 staining. At 7 DIV, DARPP-32 expression is visible in the soma but does not fill the entire cell. When DARPP-32 expression did not fill the entire neuron, EGFP signal was used for Sholl analysis. In mature MSNs, EGFP and DARPP-32 overlap to fill the entire MSN (data not shown).

### Quantification and Analysis of Dendrite Branching

Sholl analysis in Figures 1, 6 was done manually. The ImageJ Concentric Circles plugin^1^ was used to place concentric rings every 10.7 μm from the center of the soma out to ~150 μm from the cell’s center. The Cell Counter plugin^2^ was used to mark and count processes crossing each ring, starting at the third ring (21.4 μm) from the center of the cell. Replicates from two independent cultures were pooled. Neurons in Figures 4–6 were first traced using ImageJ plugins NeuronJ^3^ and quantified using XL-Calculation as previously described (Popko et al., 2009). Sholl analysis was then automated using the NeuronJ generated tracings (untraced images had too much background for reliable automated Sholl analysis) and the ImageJ plugin Sholl Analysis.^4^ In all cases, statistical analysis was conducted using GraphPad Prism (GraphPad Software). Tracing data (Figure 4C) were compared using a Mann-Whitney test (two tailed). Sholl crossing data were compared across the distance measured and between time points or treatments using a regular two way ANOVA, no repeated measures, with Sidak’s multiple comparisons *post hoc* test to determine distances from the soma at which arborization was significantly different, *p* < 0.05 was considered significant. Measurements are reported and displayed as mean ± SEM.

### Quantification and Analysis of Dendritic Spines

To examine dendritic spine development, images were collected using an Olympus ix71 microscope outfitted with a Personal DeltaVision module. EGFP filled MSNs were identified using DARPP-32 staining (for simple spine counts without morphometric analysis it was possible to use DARPP-32 staining alone, as this yielded identical spine density counts (data not shown)). Stretches of dendrites that included the terminal tip were imaged using a 100x objective. All dendrites imaged included a clear terminal tip at one end, but dendritic sections were not identified by branch number (e.g., primary, secondary). Z-stack images were taken at 0.15–0.2 μm intervals through the entire focal range and stacks were deconvolved using softWoRx software (Applied Precision) or AutoQuantX3 software (Imaris). NeuronStudio software [Mount Sinai School of Medicine, (Rodriguez et al., 2003, 2008)] was used for semi-automated spine analysis of deconvolved stacks. To make dendritic spine morphological comparisons, a trained analyst, blinded to condition, confirmed that spines identified by NeuronStudio met criteria for inclusion (e.g., observable neck connected to dendritic shaft, neck correctly attached to dendrite shaft, head diameter (HD) marker placed in center of head). Spine densities were calculated from individual segments of dendrite greater than 10 μm in length. Densities calculated from individual segments were treated as independent observations. If more than one segment was measured on a single neuron, the mean for all segments on the neuron was calculated and used as a single observation in order to avoid bias towards neurons that were particularly easy to visualize (e.g., a neuron with very bright staining that is well isolated from other DARPP32+ neurons). Final densities calculated from 3–4 independent cultures were pooled.

NeuronStudio calculated the HD and neck length (NL) for all identified spines. Paired head and neck measurements were treated as independent observations and measurements from 3–4 independent cultures were pooled. Two strategies were used to classify dendritic spines into morphological categories based on the relative relationship of observed spine head and neck measurements. The first strategy used three morphological categories mushroom, stubby, and thin based on studies of mature pyramidal neurons (Jones and Powell, 1969; Harris et al., 1992). Spines were categorized as mushroom if they had a NL to HD ratio of less than 1 and an absolute HD greater than 0.35 μm. Spines with a NL to HD ratio of greater than 2.5 were classified as thin. All other spines were classified as stubby (Penrod et al., 2011).

A second strategy was used to better define morphological categories that represent the diversity of spines found across the entire developmental spectrum. This is a more qualitative assessment that enables one to determine how morphologies at one time period change relative to those at other types, without using predetermined starting values, as in the three type categorization method. To this end, all paired spine HD and NL measurements from 7–28 DIV were pooled and the median values determined (HD = 0.35 μm, NL = 0.99 μm). Using a median split, spines were categorized as follows: (Type 1) long neck, small head (≥ median NL, ≤ median HD); (Type 2) long neck, big head (≥ median NL, ≥ median HD); (Type 3) short neck, small head (≤ median NL, ≤ median HD); (Type 4) short neck, big head (≤ median NL, ≥ median HD). Type 1 spines were further categorized in order to identify filopodia-like spines: (Type 5) extremely long neck and extremely small head (≥ 75% median NL, 1.47 μm, and ≤ 25% median HD, 0.28 μm). Following assignment into the categories, the density of each type of spine per segment length was determined.

Spine density measurements in Figure 2 were compared across time points using Kruskal-Wallis one-way ANOVA followed by Dunn’s multiple comparison test to determine significantly different distributions of spine types. Spine density measurements in Figure 5 were compared using a Mann-Whitney test (two tailed). Spine morphologies were compared across types and between time points using regular two-way ANOVA, no repeated measures, with *post hoc* Sidak’s multiple comparisons. *p* < 0.05 was considered significant. Measurements are reported as mean ± SEM.

## Results

### Developmental Changes in Dendritic Arborization

DARPP-32, which is highly expressed in mature MSNs and only weakly expressed in cortical neurons (Ouimet et al., 1984), was first detectable in the MSN soma at 7 DIV. Therefore, Sholl analysis (Sholl, 1953) was performed on DARPP-32 positive MSNs at various times in development, beginning at 7 DIV. At 7 DIV MSNs had an average of 4.7 ± 0.2 dendrites and the highest level of dendrite complexity was within ~20 μm of the soma, with an average of ~6.1 ± 0.45 crossings corresponding to the primary dendrites and 1–2 branches.

As expected, there was a significant increase in dendritic branching over time (Figure 1). Interestingly, branching occurred first distal, then proximal to the soma. From 10–13 DIV there was a significant increase in branching relatively distal to the soma (rings 5–7), followed at 13–16 DIV by a significant increase in crossings more proximal to the soma (rings 3–5). Dendritic complexity was stable after 19 DIV, with no further significant changes for the remaining period examined.

### Developmental Changes in Dendritic Spine Density and Morphology

Dendritic spine density increased progressively from 7 to 28 DIV and reached an average density of 12.77 ± 1.11 spines/10 μm by 28 DIV (Figure 2). At 7 DIV spine density was less than 1 spine/10 μm and was significantly lower than the density at 13 DIV (0.74 ± 0.08 spines/10 μm vs. 3.41 ± 0.33 spines/10 μm, *p* < 0.01). Compared to 13 DIV, spine density was increased significantly at 19 DIV (9.03 ± 0.56 spines/10 μm, *p* < 0.001) and 22 DIV (10.87 ± 0.78 spines/10 μm, *p* < 0.0001), beyond which time there was no further increase in significance compared to 13 DIV.

In order to assess morphological changes among developmentally significant time points, spines were compared at early (10 DIV), intermediate (19 DIV), and mature (28 DIV) time points: 10 DIV represents an early developmental time point when dendrite organization is simple and spine density is low; 19 DIV represents an intermediate time point when dendrite elaboration has stabilized, but spine density is still changing, and 28 DIV is a mature time point when both spine density and dendrite arborization have stabilized (Figures 1, 2). Previous research on dendritic spine morphology has used a three-group classification system (thin, mushroom, and stubby; see “Materials and Methods” Section for description) derived from measurements of mature pyramidal neurons (Jones and Powell, 1969; Peters and Kaiserman-Abramof, 1970; Harris and Stevens, 1989). In the current study, classification of dendritic spines from developing MSNs into these three groups revealed that at all time points, thin-type spines were the predominant spine type (~40–55% of total population). *Post hoc* comparisons showed that from 10–19 DIV there was a significant increase in the proportion of mushroom-type spines (10 vs. 19 DIV, *p* < 0.01), but no significant differences in spine types between 19 and 28 DIV. The increase in the proportion of mushroom-type spines from 10–19 DIV coincided with a decrease in the proportion of both thin and stubby-type spines, but the decrease was not statistically significant.

The three-type spine classification is optimized for mature pyramidal neurons. To account for the increased heterogeneity associated with development and the possibility that the range of spine NLs and HDs may differ in MSNs compared to pyramidal neurons, we developed a categorization scheme that could be used to classify the entire population of spines across all developmental time points examined (see “Materials and Methods” Section). These categories represent a wider range of morphologies and should, therefore, increase the capacity to detect developmental or experience-dependent changes in a specific morphological subpopulation of spines. Using a median split procedure, we defined five types of MSN spines based on all observed HDs and NLs (Figures 3A–D). This procedure identified individual spines by the relationship between their absolute HD and NL compared to the median and interquartile range observed in the whole data set. Splitting the population of spines along the medians of head diameter (HD = 0.35 μm) and neck length (NL = 0.99 μm) led to the definition of four primary spine types, 1–4, featuring spines with necks greater than and heads smaller than the median (type 1, ≥0.99 μm NL, ≤0.35 μm HD), spines with necks greater than and heads greater than the median (type 2, ≥0.99 μm NL, ≥0.35 μm HD), spines with necks shorter than and heads smaller than the median (type 3, ≤0.99 μm NL, ≤0.35 μm HD), and spines with necks shorter than and heads greater than the median (type 4, ≤ median NL, ≥ median HD). Some spines in the type 1 category were further classified into an extreme type 5 (≥1.47 μm NL, ≤0.28 μm HD) with NLs greater than the 75th percentile and HDs smaller than the 25th percentile. Spines with this morphology likely represent the immature spine precursor, a filopodia-like protrusion (Papa et al., 1995; Ziv and Smith, 1996; Fiala et al., 1998; Marrs et al., 2001).

There was a decrease in type 5 filopodia-like spines from 10–19 DIV (from 6.7% at 10 to 2.1% at 19 DIV), consistent with the idea that type 5 represents immature/nascent spines; however, this decrease was not statistically significant, most likely due to fact that spine heads have started to mature by 10 DIV. At early (10 DIV) and mature (28 DIV) time points, type 3 spines (relatively small heads and short necks) were the predominant spine type, where as at the intermediate (19 DIV) time, type 2 spines (relatively large heads and long necks) predominated (Figure 3E). There was a significant increase in type 2 spines at 19 DIV (10 vs. 19 DIV, *p* < 0.01; 19 vs. 28 DIV, *p* < 0.01), which was partnered with a significant transient decrease in type 3 spines (10 vs. 19 DIV, *p* < 0.05; 19 vs. 10 DIV, *p* < 0.01). There was no significant difference in spine types between 10 and 28 DIV. Combined, these findings indicate that MSN dendritic spine morphology remains heterogeneous, but alterable, throughout development and into maturity.

### Dopamine Positively Regulates Dendrite Elongation, Branching and Spine Density

These co-cultures are devoid of dopaminergic inputs, indicating that DA is not required for MSN dendritic branching or spine formation *per se*. However, given that dopaminergic innervation of the striatum and striatal expression of DA receptors begins as early as E14 in rat (roughly equivalent E12 in mouse), and the important role of DA in mature MSN physiology (Voorn et al., 1988; Schambra et al., 1994; Gerfen et al., 1995; Surmeier et al., 1996), we sought to determine whether DA plays a regulatory role in MSN development. Cultures were chronically exposed to 1 μm DA. To compensate for drug depletion by breakdown and/or cell uptake, media was supplemented with fresh drug (final concentration 1 μM DA, assuming total breakdown) every 3–4 days. However, variation of actual concentration within the dish is likely. Interestingly, even though DA was included from 4 DIV onward, branching patterns at 10 and 13 DIV were indistinguishable from controls without DA (Figure 4A). However, at 16 and 19 DIV, Sholl analysis revealed that DA treatment significantly enhanced dendritic arborization (Figure 4A).

Although Sholl analysis reveals changes in dendrite arborization, it cannot distinguish between effects on the number and/or length of dendrite branches. Neurite tracing (Figures 4B,C) revealed that although DA did not alter the number of primary dendrites, it significantly increased the length of the primary dendrites (Figure 4D), accounting in large part for the increased number of crossings at the most distal positions (rings ≥12) in the Sholl analysis. The major effect of DA on MSN arborization appears to have been on the number of dendrite branches; chronic treatment of DA resulted in an approximate 36% increase in the number of dendrite branches per neuron (Figure 4E; 14 ± 0.51 branches in controls vs. 19 ± 0.58 in the presence of DA, *p* < 0.001). The increase in the number of branches was due primarily to an increase in the number of secondary dendrites (i.e., branches off a primary dendrite, data not shown). As a result, there was a highly significant increase in the total/sum length of dendrites (Figure 4E) without major changes in the average length of each branch (data not shown).

Chronic DA caused a significant 20% increase in the density of dendritic spines at 19 DIV (Figure 5C; 10 ± 0.58 control vs. 12 ± 0.61 spines/10 μm with DA, *p* < 0.05). Whether this increase in spine density results from an increase in spine initiation and/or enhanced maintenance of spines remains to be determined.

### The Effects of BDNF on MSN Dendritic Arborization Depend on the Cellular Environment

BDNF plays an important role in the development and survival of MSNs (Baydyuk and Xu, 2014) and has been shown to significantly enhance dendritic arborization in striatal mono-culture (Rauskolb et al., 2010). In the co-cultures, chronic treatment with 10 ng/ml BDNF (a concentration of BDNF that is sufficient to induce MSN maturation in striatal mono-culture Bogush et al., 2007) caused a small, but significant increase in MSN dendritic branching proximal to the soma (Figure 6B, rings 3–4, blue line compared to untreated control, black line). Treatment of cultured hippocampal neurons with 25 ng/ml BDNF from 7–10 DIV results in a similarly small, but significant increase in proximal dendrite branching (Kwon et al., 2011). In contrast, addition of the TrkB antagonist ANA12 reduced distal MSN dendritic branching (Figure 6A, rings 7–9, 11–12). Because cortical neurons secrete BDNF, we controlled for the presence of endogenous BDNF by comparing our usual co-cultures with cultures in which the striatal and cortical neurons are grown in the same dish, but physically separated from each other (i.e., no-contact). In both conditions, the volume of media was the same; however, in co-culture and no-contact conditions, unlike striatal mono-culture, global concentrations of molecules secreted by the striatal or cortical neurons should be similar (though local concentration and secretion that is contact-dependent may vary). As previously shown for striatal mono-culture, addition of BDNF to the no-contact condition significantly enhanced dendrite branching along most of the length of the dendrites (Figure 6C, rings 3–12, blue line). These results suggest that endogenous BDNF does play a role in normal MSN development and could be interpreted as indicating that in the co-culture situation endogenous levels of BDNF within the dish are nearly saturating.

Alternatively, striatal mono-culture and our no-contact cultures lack cortical-striatal glutamatergic synapses, leading to the speculation that glutamatergic synaptic activity could regulate MSN response to BDNF. Application of the AMPA receptor antagonist CNQX had no effect on dendritic arborization (data not show); however, application of the NMDA receptor antagonist APV decreased dendritic branching in the co-culture (Figure 6B, red line), but did not alter branching in the no-contact conditions (Figure 6C, red line) or affect BDNF-induced branching in the no-contact condition (Figure 6C, green line). Compared to BDNF alone, co-application of exogenous BDNF and APV to the co-culture resulted in a significant increase in dendrite branching along much of the length of the dendrites (Figure 6B, rings 3–10, and 13, green line compared to APV alone, red line), indicating that endogenous BDNF has not reached saturating conditions. To facilitate comparisons, values for the BDNF ± APV conditions are plotted as a percentage of their respective controls (Figure 6D). This comparison demonstrates that in the absence of NMDAR activity (gray and white bars), BDNF significantly increases branching, while in the presence of NMDAR activity (black bars) the effects of BDNF are minimal. These findings reinforce the importance of excitatory synaptic contacts, identify an NMDAR-dependent signaling cascade mediating MSN development and indicate that the ability of BDNF to enhance MSN dendritic arborization is negatively regulated by NMDA receptor activity. Whether this effect is direct or indirect remains to be determined.

## Discussion

In order to understand the functional impact of dendritic complexity and discover the molecules that regulate MSN development, a time course for developmental changes in morphological complexity must first be defined. To that end, we characterized the developmental changes in MSN dendritic arborization, spine density and spine morphology in striatal-cortical culture. In concordance with previous qualitative examinations of *in vivo* MSN development, we find MSNs *in vitro* undergo significant developmental increases in dendrite complexity and dendritic spine density that are maintained in mature MSNs.

We found that in co-culture very little dendritic branching occurs before 10 DIV. The first wave of MSN dendritic branching occurs after 10 DIV and results in significant increased branching relatively distal (~50–80 μm) from the cell body. A second, more proximal, wave of dendrite branching occurs from 13–16 DIV, with significant increases in dendrite branching ~20–40 μm from the cell body. The timing of this wave of proximal branching is roughly equivalent to early postnatal stages *in vivo* where the greatest peaks in MSN branch complexity are also proximal to the soma (Rauskolb et al., 2010; Berlanga et al., 2011; Lee and Sawatari, 2011). Dendrite complexity is maximal by 19 DIV and is maintained through 28 DIV (the last time point examined).

We and others have shown that excitatory input from cortical neurons (i.e., co-culture) is necessary for MSN dendritic spine formation (Segal et al., 2003; Penrod et al., 2011) and development of *in vivo*-like electrophysiological characteristics *in vitro* (Penrod et al., 2011). Addition of glutamate or NMDA to mono-cultures does not enhance dendritic arborization or spine formation (data not shown; Segal et al., 2003). Our data indicate that excitatory input alone does not account for the enhanced dendritic arborization seen in the co-culture; even in the presence of the NMDA-R antagonist (APV), MSN dendritic arborization is significantly greater in co-culture compared to mono-culture, suggesting that the co-culture provides other factors (diffusible or contact mediated) that are important for MSN development.

A progressive increase in MSN spine density occurred from 7–19 DIV, with the largest increase occurring between 16 and 19 DIV, just after dendritic arborization was maximal. Spine density plateaued to a final average density of ~12 spines/10 μm (Figure 2), in a range previously reported for MSNs *in vivo* (Jedynak et al., 2007; Neely et al., 2007; Christoffel et al., 2011; Ding et al., 2011). Much like the case of dendrite complexity, this stably maintained spine density reflects the overall health of the MSNs *in vitro*, lending further support to the use of this system for studying MSN development.

We found that the population of MSN dendritic spines had highly variable morphologies across development, even at the most mature time points. In pyramidal neurons, a plot of spine head and neck measurements shows a developmental shift towards big heads and short necks (Papa et al., 1995) while a similar plot for MSN spines shows no such shift (Figure 3). These findings in pyramidal neurons lead to the development of specific structure-function hypotheses about dendritic spines in which spines with short necks and large heads are viewed as the most mature morphology (reviewed in Ethell and Pasquale, 2005). We were able to classify MSN spines using these categories (mushroom, thin, and stubby) and our mature MSNs had proportions of the three types similar to those reported previously *in vivo* (data not shown; DiFiglia et al., 1980; Christoffel et al., 2011); however, we believe these categories may not accurately capture the variety of morphological types seen across development, and even less so across neuronal subtypes. This conclusion is supported by a stereo electron microscopy examination of mature MSN spines *in vivo* that concluded “… no simplification of spine morphologies based on … spine types [observed in pyramidal neurons as described in Jones and Powell (1969)] … can be fit to the variation observed for neostriatal spines” (Wilson et al., 1983) and suggests that our findings of maintained morphological heterogeneity in MSN spine morphology are not *in vitro* artifacts.

Because a detailed morphological characterization of MSN dendritic spine development *in vivo* was not previously available, we utilized the large dataset we have generated from our *in vitro* studies to better describe this morphologically diverse population of spines. Using a median split procedure we generated five subtypes of MSN spines based on the functionally relevant parameters of spine HD and NL. In pyramidal neurons, spine head volume/area has been shown to correlate with post-synaptic density (PSD) size and AMPA/NMDA receptor complement (Harris and Stevens, 1989; Matsuzaki et al., 2001; Nimchinsky et al., 2002; Noguchi et al., 2005), while NL is thought to regulate diffusion of calcium (Svoboda et al., 1996; Sabatini et al., 2001; Hayashi and Majewska, 2005; Noguchi et al., 2005; Grunditz et al., 2008). The transient increase in type 2 (long neck/big head) and decrease in type 3 (short neck/small head) spines seen at 19 DIV is particularly interesting; while these types represent two extremes of HD and NL relationships, they are similar in that they represent the spine types with the strongest positive correlation between NL and HD. Because glutamatergic synapses are predominantly on the spine head and dopaminergic synapses are on the neck (Gerfen, 1988), it is tempting to speculate that the positive correlation between spine NL and HD found in mature MSNs reflects an MSN intrinsic mechanism regulating the relative accessibility for these types of synapses. Such a model further predicts that the transient peak in Type 2 spines at 19 DIV might reflect a period of increased plasticity that could provide maximum opportunity for the formation of both dopaminergic (neck) and glutamatergic (head) synapses.

Comparison of MSN growth in striatal mono- and striatal-cortical co-cultures has shown that the presence of excitatory inputs greatly enhances dendritic arborization and is essential for dendritic spine development (Segal et al., 2003; Penrod et al., 2011). Here we have shown that in co-culture DA appears to play a modulatory role in MSN development (Figures 4, 5). In striatal mono-culture, transient activation of the DA D1 receptor, but not the D2 receptors, stimulates dendritic branching as early as 7 DIV (Schmidt et al., 1996, 1998). In contrast, in co-culture the chronic application of DA did not appear to significantly enhance MSN dendritic growth until after 13 DIV. This difference could be due to the transient vs. chronic application of DA; however, we believe it more likely that the enhanced branching in the co-cultured MSN may mask the relatively smaller effects of DA on branching at earlier time points. It is also possible that in the more robustly growing co-cultures, the pathways regulating MSN dendritic branching are not responsive to DA until later in development.

Studies in striatal mono-culture have shown that chronic activation of the D1 and/or D2 DA receptors leads to a reduction in GABAergic synapse number in developing MSNs (Goffin et al., 2010), while co-culture of MSNs with dopaminergic neurons (in the absence of excitatory cortical neurons) slightly increased dendrite branching and spine formation and renders MSNs more responsive to glutamate (Fasano et al., 2013). We found that in the presence of glutamatergic input, application of DA increased dendrite branching to an even greater extent than previously shown in mono-culture, indicating that in developing MSNs activity may potentiate the morphological effects of DA. Together, these studies suggest that DA may modulate MSN development by both up-regulating dendritic branching and formation of glutamatergic spines and down-regulating GABAergic synapses. In contrast, application of DA to developing cultured cortical pyramidal neurons reduces dendrite outgrowth and branching via the D1 receptor (Li et al., 2013). These findings highlight key differences in the role of DA in the development of MSNs and pyramidal neurons. It will be interesting to determine the contributions of the different DA receptor groups in modulating MSN development in co-culture.

BDNF is produced by both cortical and mesencephalic projection neurons, but not striatal neurons (Baydyuk and Xu, 2014). CNS deletion of BDNF or its TrkB receptor during development results in decreased survival of MSNs and reduced dendritic arborization in the surviving MSNs (Baquet et al., 2004; Rauskolb et al., 2010; Baydyuk et al., 2011). In addition, application of BDNF to striatal mono-culture significantly enhances dendritic arborization (Figure 6; Rauskolb et al., 2010). However, we found that in striatal-cortical co-culture, the ability of exogenous BDNF to enhance MSN dendritic arborization appeared to be negatively regulated by activity of the NMDA receptor. This finding supports the idea that parallel, but potentially intersecting, signaling pathways mediate MSN development, one acting through NMDAR activation in MSNs and another through BDNF signaling to MSNs. In situations where the NMDAR-dependent pathway has been reduced (via APV introduction, mono-culture or no-contact), exogenous BDNF is able to significantly increase branching.

The current experiments cannot conclusively determine whether BDNF and APV are acting directly on the MSNs or indirectly via cortical glutamatergic neurons or other interneurons or glia in the culture. For example, it is possible that in the co-culture APV serves to reduce MSN and cortical neuron activity. Since NMDAR activity regulates cortical release of BDNF (Park et al., 2014), APV could reduce release of endogenous BDNF, leaving room for exogenous BDNF to be effective. However, if the effect of APV was due primarily to reduced BDNF release by pyramidal neurons, then one would expect APV and the TrkB antagonist ANA12 to reduce MSN dendritic arborization to similar extents, which is not the case (Figure 6). Furthermore, exogenous BDNF significantly increased MSN arborization in both the APV-treated and no-contact conditions, consistent with the conclusion that BDNF is acting directly on the MSNs. The fact that APV only affects MSN dendritic arborization in the co-culture further suggests that this effect is due to APV inhibition of cortico-striatal synapse. Finally, it is also possible that exogenous BDNF application is positively regulating cortical release of glutamate (Madara and Levine, 2008), and that the effects of exogenous BDNF are due in part to increased release of glutamate. However, the addition of exogenous glutamate or NMDA to the cultures does not enhance MSN dendritic arborization (data not shown) or spine formation (Segal et al., 2003), suggesting that cell-cell contact (e.g., synaptic contact) is required.

Our *in vitro* findings may impact interpretation of *in vivo* studies. Given the fact that BDNF also regulates arborization of cortical neurons (Gorski et al., 2003), it possible that depletion of BDNF/TrkB could lead to reduced activity of excitatory cortical-striatal afferents. Reduced excitatory input during development could, in turn, lead to reduced MSN dendritic arborization *in vivo* just as addition of APV reduces MSN arborization *in vitro*. MSNs with reduced excitatory input *in vivo* might be especially sensitive to exogenous BDNF, similar to MSNs *in vitro*. This leads to the prediction that pathological situations that reduce cortical-striatal activity might render MSNs especially sensitive to “rescue” by exogenous BDNF.

Results from the current analysis of MSN development *in vitro* are similar to previous qualitative descriptions of postnatal development of MSN *in vivo* (DiFiglia et al., 1980; Hull et al., 1981; Rafols et al., 1989) and the quantitative analysis of mature MSN spines *in vivo* (Wilson et al., 1983), indicating that the development of MSNs in striatal-cortical culture likely recapitulates many key aspects of MSN development *in vivo*. The observation that the nature of the cultures significantly alters MSN response to signals such as BDNF further emphasize the advantage of studying MSN development in a co-culture system. Future studies in this system can address intrinsic and extrinsic factors affecting MSN development and determine how these factors affect functional plasticity.

## Conflict of Interest Statement

The authors declare that the research was conducted in the absence of any commercial or financial relationships that could be construed as a potential conflict of interest.

## Footnotes

^1^http://rsbweb.nih.gov/ij/plugins/concentric-circles.html

^2^http://rsbweb.nih.gov/ij/plugins/cell-counter.html

^3^http://www.imagescience.org/meijering/software/neuronj/

^4^http://fiji.sc/Sholl_Analysis
